# Genes of the *de novo* and Salvage Biosynthesis Pathways of Vitamin B6 are Regulated under Oxidative Stress in the Plant Pathogen *Rhizoctonia solani*

**DOI:** 10.3389/fmicb.2015.01429

**Published:** 2016-01-05

**Authors:** Jamil Samsatly, Rony Chamoun, Emile Gluck-Thaler, Suha Jabaji

**Affiliations:** ^1^Department of Plant Science, McGill UniversitySte-Anne-de-Bellevue, QC, Canada; ^2^Department of Plant Pathology, Ohio State UniversityColumbus, OH, USA

**Keywords:** vitamin B6, *de novo* pathway, salvage pathway, *Rhizoctonia solani*, oxidative stress, abiotic stress, *PDX* genes, antioxidant genes

## Abstract

Vitamin B6 is recognized as an important cofactor required for numerous metabolic enzymes, and has been shown to act as an antioxidant and play a role in stress responses. It can be synthesized through two different routes: salvage and *de novo* pathways. However, little is known about the possible function of the vitamin B6 pathways in the fungal plant pathogen *Rhizoctonia solani*. Using genome walking, the *de novo* biosynthetic pathway genes; *RsolPDX1* and *RsolPDX2* and the salvage biosynthetic pathway gene, *RsolPLR* were sequenced. The predicted amino acid sequences of the three genes had high degrees of similarity to other fungal PDX1, PDX2, and PLR proteins and are closely related to other *R. solani* anastomosis groups. We also examined their regulation when subjected to reactive oxygen species (ROS) stress inducers, the superoxide generator paraquat, or H_2_O_2_, and compared it to the well-known antioxidant genes, *catalase* and *glutathione-S-transferase* (*GST*). The genes were differentially regulated with transcript levels as high as 33 fold depending on the gene and type of stress reflecting differences in the type of damage induced by ROS. Exogenous addition of the vitamers PN or PLP in culture medium significantly induced the transcription of the vitamin B6 *de novo* encoding genes as early as 0.5 hour post treatment (HPT). On the other hand, transcription of *RsolPLR* was vitamer-specific; a down regulation upon supplementation of PN and upregulation with PLP. Our results suggest that accumulation of ROS in *R. solani* mycelia is linked to transcriptional regulation of the three genes and implicate the vitamin B6 biosynthesis machinery in *R. solani*, similar to *catalases* and *GST*, as an antioxidant stress protector against oxidative stress.

## Introduction

Vitamin B6 is a collective term that refers to a group of six vitamers: pyridoxal (PL), pyridoxine (PN), pyridoxamine, and their phosphorylated derivatives (PLP, PNP, PMP) (Fitzpatrick et al., 2012; Vanderschuren et al., 2013). In plants, fungi and prokaryotes, vitamin B6 vitamers are produced via the *de novo* biosynthetic pathway that ultimately leads to the synthesis of the most active form pyridoxal 5′-phosphate (PLP) via a heterodimer complex made up of two pyridoxal biosynthesis proteins that belong to highly conserved protein families (PDX1 and PDX2) (Mittenhuber, 2001; Raschle et al., 2005; Fitzpatrick et al., 2007). Of all the vitamers, PLP is critically important because it is essential as a cofactor for over 140 chemical reactions (Percudani and Peracchi, 2003; Roje, 2007; Hellmann and Mooney, 2010). In addition to the *de novo* pathway, a conserved “salvage pathway” is found in all organisms (González et al., 2007; Herrero et al., 2011; Rueschhoff et al., 2012). Reactions in the salvage pathway include the reduction of PL to PN, which is carried out by pyridoxal reductase (PLR), a downstream enzyme in the vitamin B6 biosynthesis pathway (Morita et al., 2004; Herrero et al., 2011), and the conversion of PN into PNP which is performed by PNP oxidase generating at the end of its pathway PLP (González et al., 2007; Sang et al., 2007).

In recent years, vitamin B6 has been identified as a potent antioxidant with a high ability to quench reactive oxygen species (ROS), resulting in an antioxidant capacity that rivals that of tocopherols or ascorbic acid, and may play a role in stress responses in fungi and plants (Mooney et al., 2009; Vanderschuren et al., 2013). The antioxidant properties of vitamin B6 was originally reported in the fungal pathogen *Cercospora nicotianae*, by providing resistance to cercosporin, a singlet oxygen generating toxin (Ehrenshaft et al., 1999; Bilski et al., 2000; Daub and Ehrenshaft, 2000). This novel characteristic of vitamin B6 as a ROS scavenger and its ability to increase resistance to biotic and abiotic stresses have been demonstrated in plant-microbe interaction studies (Danon et al., 2005; Denslow et al., 2005). Supplementation with PM could delay or decrease pathogen-induced leaf necrosis (Denslow et al., 2005) while PN could protect Arabidopsis *flu* mutant, which releases singlet oxygen in plastids, from cell death (Danon et al., 2005).

Studies on vitamin B6 metabolism and regulation are limited to a few fungi, (Ehrenshaft et al., 1999; Osmani et al., 1999; Benabdellah et al., 2009) including one plant pathogenic fungus, *C. nicotianae*. Necrotrophic fungi are successful pathogens that are able to overcome or suppress an array of complex ROS-mediated plant defenses (Chung, 2012). The relative sensitivity of necrotrophic plant pathogens to ROS is likely determined by the effectiveness of their own ROS detoxification ability. To survive under aerobic conditions, fungi must possess detoxification systems such as NADPH oxidase (NOX) complex that can effectively scavenge ROS, maintain reduced redox states within subcellular microenvironments, and repair ROS-triggered damage (Heller and Tudzynski, 2011; Chung, 2012).

The soil fungus *Rhizoctonia solani* Kühn (teleomorph *Thanatephorus cucumeris*, Frank, Donk) is a generalist, necrotrophic pathogen with a wide host range, causing damping-off of seedlings, root crown, stem rots, and sheath blight diseases of plants (Ogoshi, 1996; Sneh, 1996). Basal resistance to Rhizoctonia diseases in several crops is correlated with ROS-scavenging mechanisms such as hydrogen peroxide (H_2_O_2_) production, enhanced peroxidases (POX), and superoxide dismutase (SOD) activities, transcriptional regulation of NOX, regulation of several metabolites in the phenylpropanoid and vitamin B6 biosynthetic pathways, accumulation of oxidized fatty acids, and increased levels of cell wall bound phenolics (Taheri and Tarighi, 2011; Aliferis and Jabaji, 2012; Foley et al., 2013; Nikraftar et al., 2013; Aliferis et al., 2014). However, data on ROS-scavenging systems in *R. solani* is very limited. To date, evidence that *R. solani* genes of the vitamin B6 pathway are upregulated in response to biotic stress has been reported (Morissette et al., 2008; Chamoun and Jabaji, 2011; Gkarmiri et al., 2015). Parasitized hyphae and sclerotia of *R. solani* by the mycoparasite *Stachybotrys elegans* have displayed a substantial up-regulation in the transcription of the gene encoding PLR (Chamoun and Jabaji, 2011). Other vitamin B6 biosynthetic encoding genes such as pyridoxal-5-phosphatases and transaminases were recently reported to be upregulated in *R. solani* in response to antagonistic plant associated bacteria (Gkarmiri et al., 2015).

With the aim of gaining insight into the possible implication of ROS on vitamin B6 regulation in *R. solani*, we report on the characterization of three vitamin B6 genes, *RsolPDX1*, and *RsolPDX2* from the vitamin B6 *de novo* pathway and *RsolPLR* from the salvage pathway. The characterized genes are homologs of *R. solani* and other known fungal vitamin B6 genes. *R. solani* vitamin B6 *de novo* pathway genes were up-regulated by the superoxide generator paraquat but not by H_2_O_2_ whereas *RsolPLR* was up-regulated by both chemicals. This displays a unique response of these genes according to the type of oxidative stress induced by ROS generating chemicals.

## Materials and methods

### Fungal strains, media, and culture conditions

Starter culture of *R. solani* Kühn AG3 (isolate Rs114, ATCC 10183) was grown on potato dextrose agar (PDA; Difco, Detroit, USA) at 24°C for 5 days. For the isolation of vitamin B6 related genes (*RsolPDX1, RsolPDX2, RsolPLR*) from *R. solani*, plugs (5 mm) from starter cultures were placed in the center of fresh PDA plates, overlaid with cellophane membranes (500 PUT; UCB, North Augusta, USA), and grown for 7 days at 24°C. Cultures of *Fusarium oxysporum* (ATCC 60860) and *Trichoderma virens* (DAOM 169262) were grown similarly to *R. solani* and used as controls.

### Experimental setup for oxidative stress

To investigate the role of *R. solani* in detoxification/homeostasis of ROS, the relative transcript abundance of vitamin B6 genes (*RsolPDX1, RsolPDX2*, and *RsolPLR*) was monitored over time when *R. solani* was subjected to different oxidative stress inducers or when exogenous additions of vitamin B6 vitamers were added. For this purpose, *R. solani* plugs (5 mm) were grown in Petri plates containing 15 mL of half-strength potato dextrose broth (PDB) (PDB; Difco, Detroit, USA) for 3 days at 24°C. Subsequently, the PDB media in *R. solani* cultures was removed and substituted with 15 mL of fresh PDB amended with one of the following stress inducers: 5 mM of H_2_O_2_ (Sigma, Toronto, ON, Canada), 7.5 mM of phenylacetic acid (PAA) (Sigma) and 4 mM of the superoxide generator paraquat (N,N′-dimethyl-4,4′-bipyridinium dichloride), (Syngenta Crop Protection, Toronto, ON, Canada), or with the vitamin B6 vitamers: 0.01 g L^−1^ pyridoxine (PN) (Sigma), and 0.01 g L^−1^ pyridoxal-5′-phosphate (PLP) (Sigma). No amendment was added to the half-strength PDB media in the control plates. Oxidant sensitivity and effective concentrations (EC_50_) for H_2_O_2_ and paraquat were determined by obtaining dose-response curves and the concentration which resulted in near 50% inhibition of *R. solani* growth at 72 hours post treatment (HPT) (Wang et al., 2011), whereas the concentration of applied PAA was selected based on previous reports (Bartz et al., 2012). The vitamin B6 vitamers were chosen based on optimized concentrations that do not inhibit the growth of *R. solani*.

Comparison of the relative transcript abundance of *RsolPDX1, RsolPDX2*, and *RsolPLR* genes with the well-established antioxidants *glutathione S-transferase* (*GST*) and *catalase* genes was performed using specific primers designed in this study (Table 1). All experiments were conducted with three biological replicates and two technical replicates per treatment or control. Mycelia from treatments and controls were harvested at different HPT that spanned from 0.5 to 72 h depending on the type of the stress inducer, flash-frozen in liquid nitrogen, and processed for RNA extraction. Additional culture plates of *R. solani* exposed to H_2_O_2_ and paraquat were kept for light and fluorescence microscopy.

**Table 1:** **List of primers of ***R. solani*** used in this study**.

**Primer**	**Sequence (5′ → 3′)**	**Annealing temperature (°C)**	**Amplicon size (bp)**	**Method**	**References**
**VITAMIN B6** ***de novo*** **PATHWAY GENES**					
DegF-(*PDX1*)	AAGGTACCTGTNACVATYCCNGTBATGG	54	670	Degenerate PCR	This study
DegR-(*PDX1*)	TTCTGCAGAGCNGCRTCVGCVGGNGTVGC				
5′GSP1-(*PDX1*)	TGACGAACAGCCTCGACAACATTTCC	62	219	Genome walker and Real time-PCR	This study
3′GSP2-(*PDX1*)	TCCGTTTGTCTGTGGGGCTACATCTCTC				
5′GSP2-(*PDX1*)	ATCATGGCTGCGCCTTCGGAAATACG	62	–	Genome walker	This study
3′GSP1-(*PDX1*)	TCTCACCCCTGCTGACGAACAGCATC	62	300	Genome walker and Southern blot	This study
*PDX1*probeR	TTGCGAATCTCGGCGTTGACCG	65		Southern blot	
DegF-(*PDX2*)	AACTGCAGTTGGGGNACHTGYGCNGG	51	260	Degenerate PCR	This study
DegR-(*PDX2*)	CCTCTAGAGACNGGNGCNCKDATRAA				
3′GSP2-(*PDX2*)	AAGAAGGGTGGTCAAGAGGTTTTTGG	72	–	Genome walker	This study
5′GSP2-(*PDX2*)	CGAAAGATTCAAGCTTCAATGGTGCTTA				
3′GSP1-(*PDX2*)	AATCTTGCTTGCCTCTGGTGGTGTTG	72	225	Genome walker, Real time-PCR	This study
5′GSP1-(*PDX2*)	ATCCCATTAAATGGTCGGTCCTCATCA	72	640	Genome walker, Real time-PCR and Southern blot	
*PDX2*probeF	ATGACTAGAACTGAAACGGAAC	62		Southern blot	
**VITAMIN B6 SALVAGE PATHWAY GENES**					
*PLR* AKR8 F	GAAAGCCTCCTCTTGGAATCT	58	300	Real time-PCR and Southern blot	Chamoun and Jabaji, 2011
*PLR* AKR8 R	GGGTAAGATTGGATCGATTGGG				
5′GSP1-(*PLR*)	GGCGATGATCTTTAATGCGTCCACTAG	67	–	Genome walker	This study
5′GSP2-(*PLR*)	TTTGAAAGCCTCCTCTTGGAATCTGG				
3′GSP1-(*PLR*)	AAGTTTCATTCTGGAGCTACGAGGAAG				
3′GSP2-(*PLR*)	TAAAGTGATTGCCAAGGCTGCTGAAATTG				
5′GSP3-(*PLR*)	TAATAGAAAGCAAGAAATCGC	52	837	Sequencing	This study
3′GSP3-(*PLR*)	GCTCAAATAAATCAACCTTC				
5′GSP4-(*PLR*)	AAGAGACTCGTAAAGGTGCG	57	791	Sequencing	This study
3′GSP4-(*PLR*)	ATGCCACCAATTTCGTTTCAG				
**ANTIOXIDANT GENES**					
*RS-GST*^a^*-F*	AGAAGACGAGGCAAATGCGA	57	256	Real time-PCR	gi|576992090|
*RS-GST*^a^*-R*	ATCTCTTCAACCGCCTTCCAGT				
*RS-Catalase-F*	ACCAGAAGTGTTAGTCCAGCGG	56	190	Real time-PCR	gb|JATN01000310.1|
*RS-Catalase-R*	CATCCGGTCACAGCAGCGTAA				
**REFERENCE GENES**					
*Tubulin-F*	GTTGATTTCCAAGATCCGTG	55	139	Real time-PCR	FJ392707
*Tubulin-R*	CGAGTTCTCGACCAACTGAT				
*Histone 3-F*	AAGTCTGCACCCGTAAGTTC	55	289	Real time-PCR	M. Cubeta^b^
*Histone 3-R*	AACAACGAGACGAGGTAAGC				
*G3PDH^c^-F*	GGTATTATTGGATACACTGA	55	129	Real time-PCR	Chamoun and Jabaji, 2011
*G3PDH^c^-R*	TTAAGCCTCAGCGTCTTTCT				
ITS1-F	CTTGGTCATTTAGAGGAAGTAA	55	Variable	cDNA quality	Gardes and Bruns, 1993
ITS4-R	TCCTCCGCTTATTGATATGC				

a *Glutathione S-Transferase*.

b *Sequence of Histone 3 gene was provided by Marc Cubeta, North Carolina State University*.

c *G3PDH: Glyceraldehyde-3-phosphate dehydrogenase gene*.

### Nucleic acids extraction

Total genomic DNA was isolated from 100 mg of ground tissue of *R. solani, F. oxysporum*, and *T. virens* using the DNeasy Plant Mini Kit™ (Qiagen, Toronto, ON, Canada). Total RNA was isolated from 100 mg of ground *R. solani* mycelia exposed to various stress treatments and from the control using the RNeasy Plant Mini Kit™ (Qiagen) and treated with RNase-free DNaseI™ (Qiagen) according to the manufacturer's recommendations. The concentration and purity of RNA were assessed by spectrophotometry with ND1000 (NanoDrop, Wilmington, Delaware), while RNA quality was verified on 1.2% (w/v) formaldehyde-agarose gel electrophoresis. A total of 500 ng RNA was reverse transcribed using the Quantitect Reverse transcriptase kit™ (Qiagen).

### Genome library construction and manipulation of the vitamin B6 biosynthesis genes *RsolPDX1, RsolPDX2*, and *RsolPLR*

**Figure 1:**
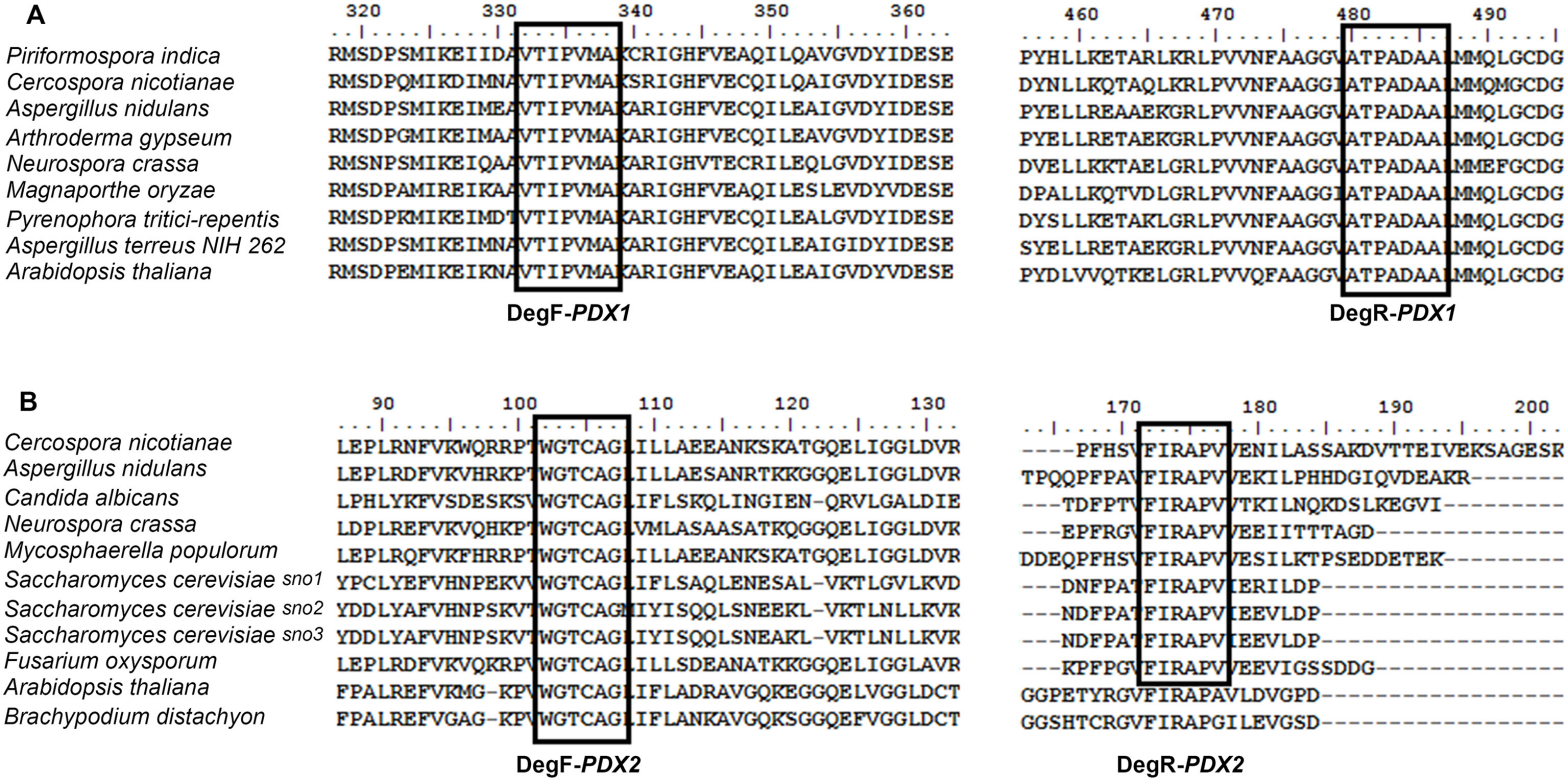
**Amino acid sequence alignment of the PDX1 (A) and PDX2 (B) proteins belonging to various fungi and plants**. The GenBank accession numbers of the PDX1 proteins **(A)** are as follows: *Piriformospora inidica* (CAFZ01000024), *Cercospora nicotianae* (AF0356191825), *Emericella nidulans* (AF133101.1), *Arthroderma gypseum* (315049582), *Neurospora crassa* (AAK07850.1), *Magnaporthe oryzae* (389628343), *Pyrenophora tritici-repentis* (189190153), *Aspergillus terreus*NIH262 (115433602), *Arabidopsis thaliana* (145360746). The GenBank accession numbers of the PDX2 proteins **(B)** are as follows: *Cercospora nicotianae* (AF294268.1), *Aspergillus nidulans* (AF363613.1), *Candida albicans* (68468776), *Neurospora crassa* (12802355), *Mycosphaerella populorum* (gi 453085089), *Saccharomyces cerevisiae* Sno1p (296147212), *Saccharomyces cerevisiae* Sno2p (296147449), *Saccharomyces cerevisiae* Sno3p (296144372), *Fusarium oxysporum* (gi 475662976), *Arabidopsis thaliana* (30697380), *Brachypodium distachyon* (357113803). The multiple sequence alignment was done using BioEdit v7.2.0. Identical amino acid residues in all the sequences are highlighted DegF: Degenerate forward primer, DegR: Degenerate reverse primer. Boxes represent the location of degenerate primers used in PCR.

Degenerate primer pairs, DegF-(*PDX1*)/DegR-(*PDX1*), and DegF-(*PDX2*)/DegR-(*PDX2*), were designed from the alignment of eight protein sequences of PDX1 (Figure 1A) and 11 protein sequences of PDX2 (Figure 1B) belonging to different fungi, respectively. Primers design was based on the conserved domains in PDX1 and PDX2 (Figures 1A,B). The primer pairs were also tested on the genomic DNA of *F. oxysporum* (ATCC 60860), and *T. virens* (DAOM 169262) since both fungi are known to harbor both genes, PDX1 (*F. oxysporum* Genbank accession number ENH63312) and PDX2 (*T. virens* Genbank accession number EHK18113), respectively. The degenerate primers were used in PCR reactions to amplify putative products of *PDX1* and *PDX2* from genomic DNA of *R. solani*.

For the construction of Genome-walking libraries, *R. solani* AG3 (Rs114) genomic DNA was digested with four restriction enzymes (EcoRV, DraI, PvuII, and StuI) following the manufacturer's recommendations (Clontech, CA, USA). For Genome-walking PCR reactions, a set of gene-specific primers (*PDX1*-GSP, *PDX2*-GSP, *PLR*-GSP: 5′GSP1, 5′GSP2, 3′GSP1, and 3′GSP2; Table 1) were designed based on the genomic DNA sequence of the putative products for *PDX1* and *PDX2*, and on *R. solani* mRNA sequence (Genbank accession number EU008744.1) for *PLR*. Specific PCR fragments were obtained, purified using the QIAquick PCR Purification Kit (Qiagen), and sub-cloned in pDrive cloning vector (Qiagen). Positive clones were sequenced and assembled in a contig using BioEdit v7.2.0 (Hall, 1999) and then blasted on NCBI for homology and identity confirmation. The full sequences of *RsolPDX1, RsolPDX2*, and *RsolPLR* genes were annotated and submitted to GenBank database (KF620111, KF620112, and KJ395592), respectively.

### Genomic southern hybridization

Specific probes for each of the *RsolPDX1, RsolPDX2*, and *RsolPLR* genes were constructed by amplifying genomic DNA fragments of *R. solani* using an appropriate set of primer pairs (Table 1). The amplified products were DIG-labeled using the DIG-High Prime DNA Labeling and Detection Starter Kit I (Roche Applied Science, QC, Canada). The genomic DNA of *R. solani* was digested with an appropriate set of restriction enzymes [(EcoRI, PstI, and HindIII for *PDX1*), (EcoRI, PstI, and KpnI for *PDX2*), (EcoRI, PstI, HindIII, and XhoI for *PLR*)], electrophoresed on a 1% agarose gel, transferred onto Nylon Hybond N^+^ membrane (Roche Applied Science), and UV cross-linked. Hybridization was performed following the procedure of Chamoun et al. (2013).

### Phylogenetic analysis

Multiple sequence alignments of *RsolPDX1, RsolPDX2*, and *RsolPLR* cDNA with other members of *PDX1, PDX2*, and *PLR* from selected fungi (Ascomycetes and Basidiomycetes) and plants were conducted using Clustal Omega (Sievers and Higgins, 2014). The tree was constructed using MEGA 6.0 and generated by the maximum likelihood method based on the General Time Reversible model with 1000 Bootstrap replicates (Tamura et al., 2011).

### Quantitative RT-PCR

QRT-PCR assays were conducted on five target genes *RsolPDX1, RsolPDX2, RsolPLR, GST*, and *catalase*, and three reference genes *G3PDH, Histone-3*, and β*-Tubulin* (Table 1) using Stratagene Mx3000 (Stratagene, Cedar Creek, USA). Each template and negative control had three biological replicates with two technical replicates included in each run. PCR assays were performed as previously described (Chamoun et al., 2013) using the appropriate annealing temperature for each primer pair (Table 1). The relative transcript abundance levels of the genes were calculated according to (Zhao and Fernald, 2005) and normalized against the reference genes showing the lowest minimal variation and coefficient of variation (CV) across all treatments. The relative transcript abundance of the genes was tested for significance between treatments and controls at each harvesting time point by Two-way analysis of variance (ANOVA) and, when appropriate, for least significant differences (LSD) at *P* < 0.05 using the SPSS statistical package v. 22.0, (IBM Corp., Armonk, NY, USA).

### Optical and fluorescence microscopy

To associate changes in the transcript abundance of the genes with the ROS accumulation in *R. solani* to the different stress inducers, control and treated-mycelia with H_2_O_2_ (5 mM) or paraquat (4 mM) were viewed under a light and fluorescent microscope at 3 and 24 HPT. Images were digitally documented with the Moticam 2300 digital camera (GENEQ Inc. Montreal, Quebec) for light microscopy. For ROS detection, *R. solani* mycelia were incubated with 10 μM of 2′,7′-dichlorodihydrofluorescein diacetate (H2DCF-DA), a specific ROS molecular-detection probe, in half-strength PDB for 30 min. Mycelia were then washed with pre-warmed (28°C) half-strength PDB for 30 min to remove the non-internalized probe and were examined under Zeiss SteREO Discovery.V20 fluorescence microscope (Carl Zeiss Canada Ltd., Toronto, Ontario, Canada) at 3 and 24 HPT. Fluorescence intensity emitted from individual cells was measured using an excitation wavelength of 470 nm.

## Results

### *R. solani* genes *RsolPDX1, RsolPDX2*, and *RsolPLR* are members of the vitamin B6 biosynthetic pathway

**Figure 2:**
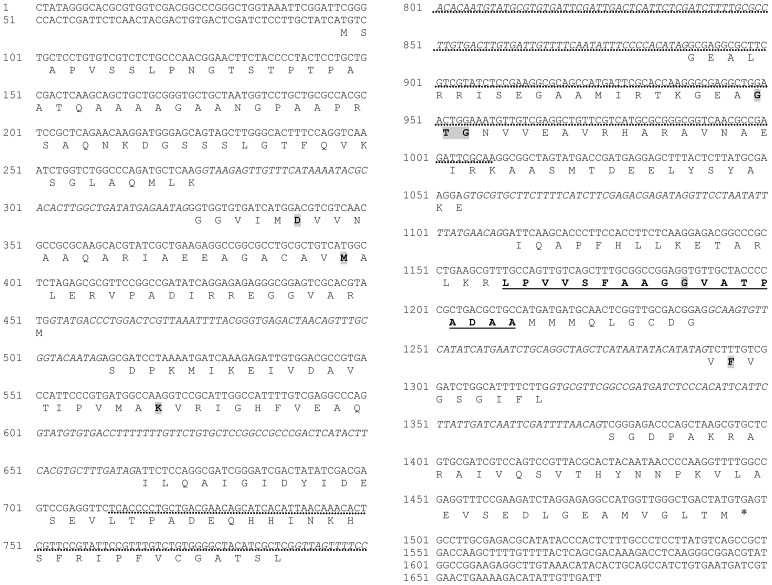
**Complete sequence of ***RsolPDX1*** (gene accession number KF620111)**. Introns are written in italic in the gene sequence. The signature motif of the PDX1 protein family is underlined. Amino acids known to be essential for enzyme activity are shadowed. Asterisk indicates end codon. Dotted line indicates southern blot probe.

**Figure 3:**
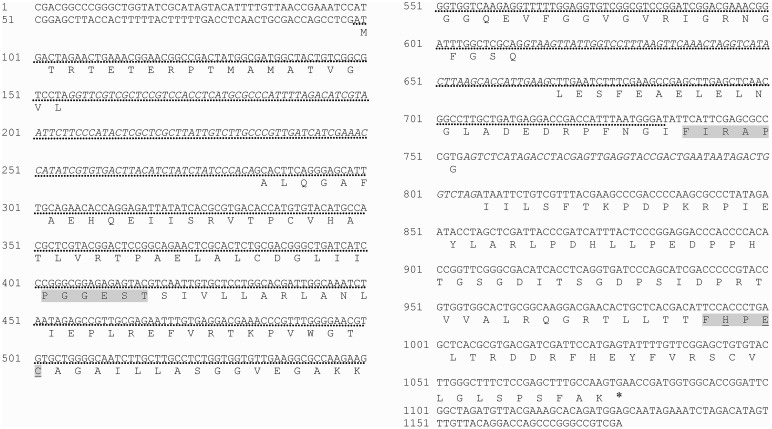
**Complete sequence of ***RsolPDX2*** (gene accession number KF620112)**. Introns are written in italic in the gene sequence. The signature motif of the PDX2 protein family C-H-E (known to be essential for enzyme activity) is underlined. Conserved amino acids are shadowed. Asterisk indicates end codon. Dotted line indicates southern blot probe.

**Figure 4:**
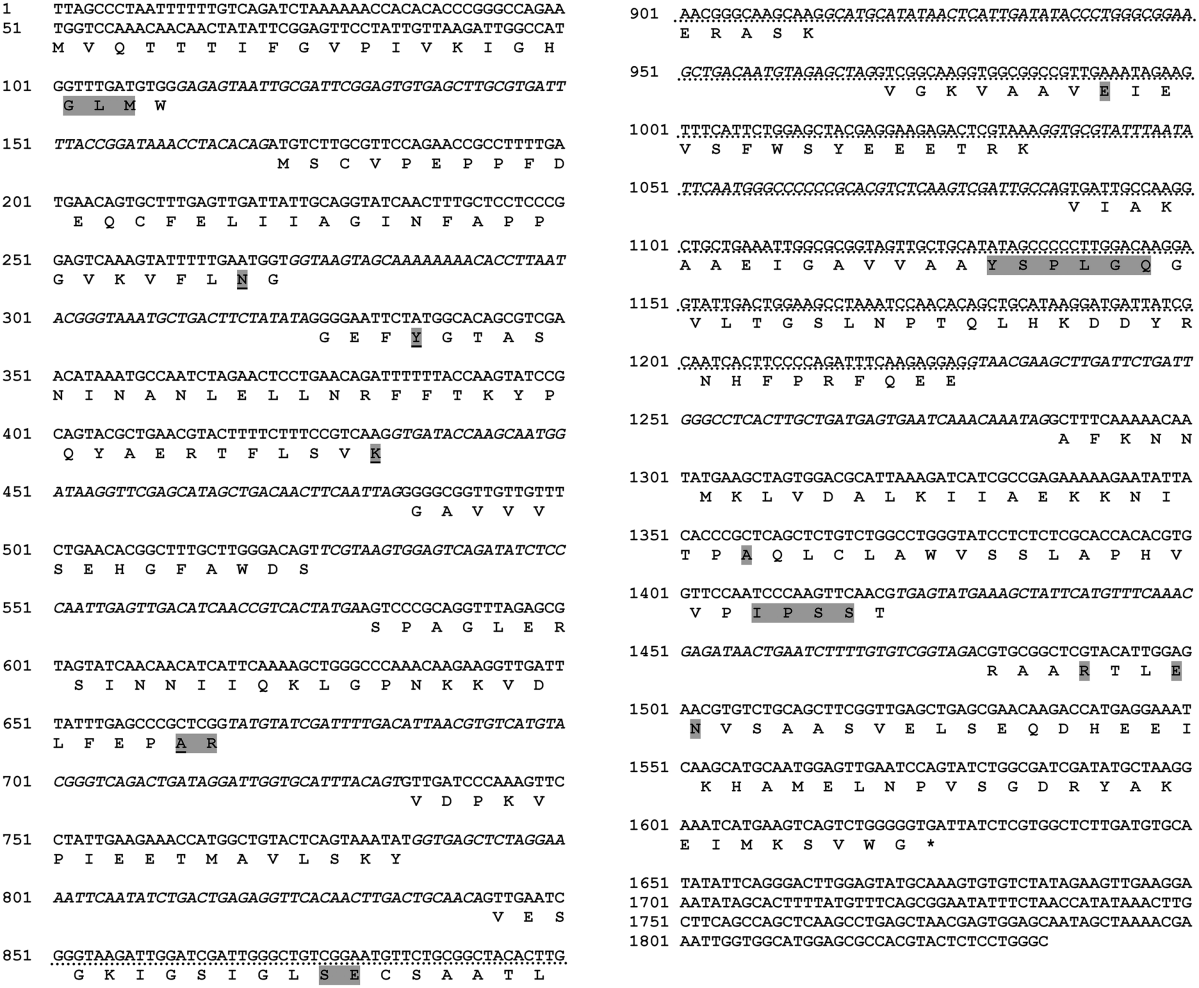
**Complete sequence of ***RsolPLR*** (gene accession number KJ395592)**. Introns are written in italic in the gene sequence. The four residues that compose the Aldo_keto_reductase conserved catalytic tetrad are underlined. Conserved amino acids are shadowed. Asterisk indicates end codon. Dotted line indicates southern blot probe.

The designed primer pairs DegF/R-(*PDX1*) and DegF/R-(*PDX2*) successfully amplified PCR products of 671 and 263 bp in size, respectively which were confirmed by sequencing to be *PDX1* and *PDX2*. Subsequently, the gene specific primers (*PDX1*-GSP, *PDX2*-GSP, *PLR*-GSP) (Table 1) were used to obtain the whole sequences of *RsolPDX1, RsolPDX2*, and *RsolPLR* using the Genome-walking technique. Initially, only partial sequence was obtained for *PLR*, hence, additional *PLR*-GSP primers (Table 1) were designed based on genome sequence of *R. solani* AG3 (strain Rhs1AP) to get the complete sequence. PCR products for each of the three genes were assembled into contigs and their sequence analysis led to the identification of the vitamin B6 genes belonging to the *de novo* pathway in *R. solani, RsolPDX1* (accession number KF620111), and *RsolPDX2* (accession number KF620112), and to a identification of *RsolPLR* (accession number KJ395592) belonging to the vitamin B6 salvage pathway. The organization of the three genes and their features are depicted in Figures 2–**4**.

The full length of the *RsolPDX1* and *RsolPDX2* sequences obtained during our analyses are 1640 and 1178 bp, respectively. The open reading frame of *RsolPDX1* consists of 1402 nucleotides encoded from 61 to 1463 bp, while the ORF of *RsolPDX2* consists of 980 nucleotides encoded from 99 to 1079 bp. Both genes are interrupted with several introns (Figures 2, 3). The predicted protein sequences of *RsolPDX1* and *RsolPDX2* consist of 322 and 248 amino acid residues with a molecular weight of 33.65 or 26.78 kDa and a calculated isoelectric point (pI) of 6.625 or 5.815, respectively. The signature motif of the PDX1 protein family (LPVVSFAAGGVATPADAA) and conserved amino acids (D, M, K, and GTG) were identified in PDX1 (Figure 2). Similarly, the signature motif of the PDX2 protein family (C-H-E) which is essential for the protein activity in addition to other conserved amino acids (i.e., PGGEST, FIRAP, and FHPE) are highlighted in PDX2 (Figure 3).

The obtained full length of the *RsolPLR* sequence is 1837 bp and its sequence analysis revealed that it belongs to the aldo_keto_reductase family. *RsolPLR* ORF is encoded from 50 to 1627 bp and is interrupted by several introns whereas conserved amino acids (i.e., AR, SE, E, and YSPLG) in PLR proteins are highlighted (Figure 4). *RsolPLR* gene encodes a putative polypeptide of 337 amino acid residues with a molecular weight of 36.92 kDa and a calculated isoelectric point (pI) of 5.89 (Figure 4).

### Gene copies of *RsolPDX1, RsolPDX2*, and *RsolPLR*

**Figure 5:**
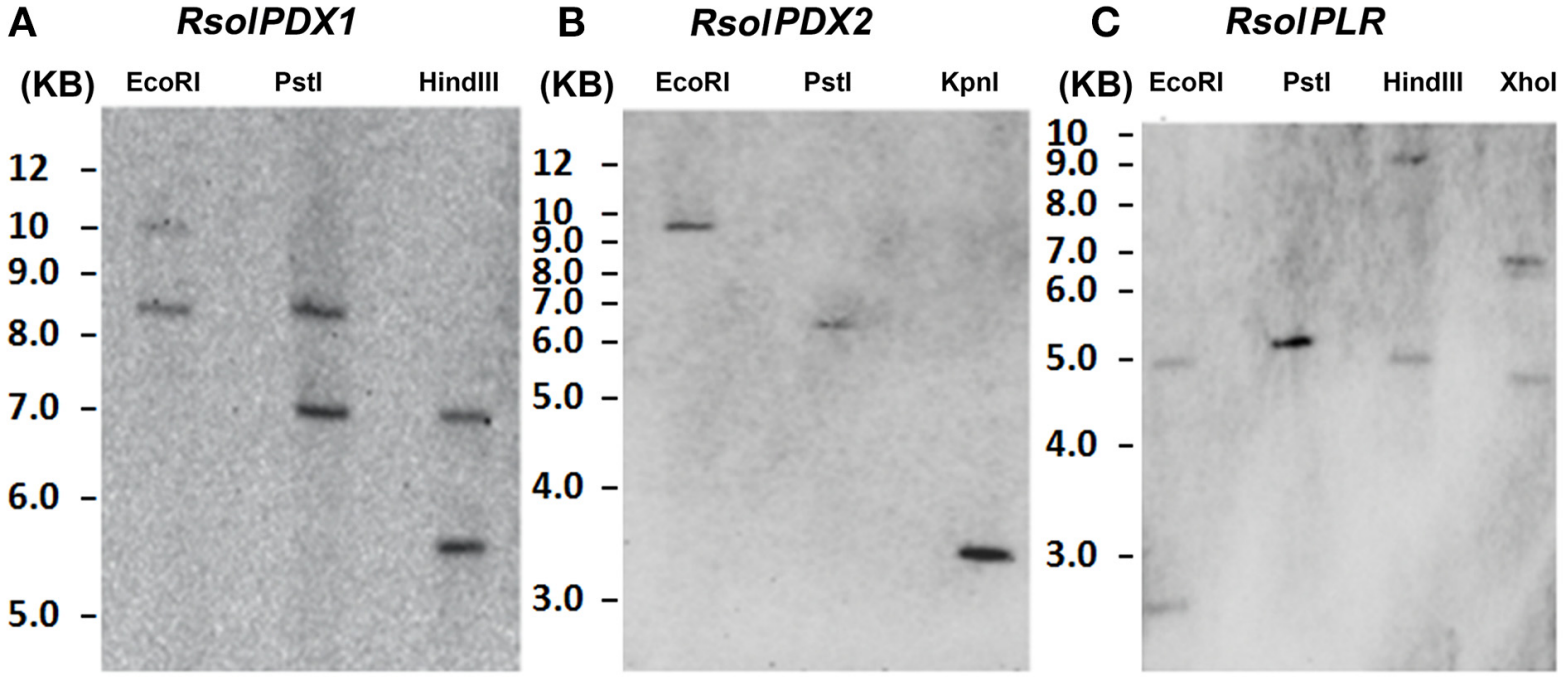
**Southern blot analysis showing gene copy number of ***RsolPDX1*** (A), ***RsolPDX2*** (B), and ***RsolPLR*** (C) in ***R. solani*** AG3**.

Gene copies of *RsolPDX1, RsolPDX2*, and *RsolPLR* in the genome of *R. solani* were estimated by Southern blot analysis (Figure 5). Under high stringency conditions of hybridization, *RsolPDX1*, and *RsolPLR* probes hybridized to more than one high molecular fragment, indicating that two copies are present for each of the genes. On the other hand, *RsolPDX2* is shown to be present as single copy as revealed by the hybridization of its corresponding probe to a single fragment (Figure 5).

### *RsolPDX1, RsolPDX2*, and *RsolPLR* are phylogenetically related to homologs of the vitamin B6 genes

**Figure 6:**
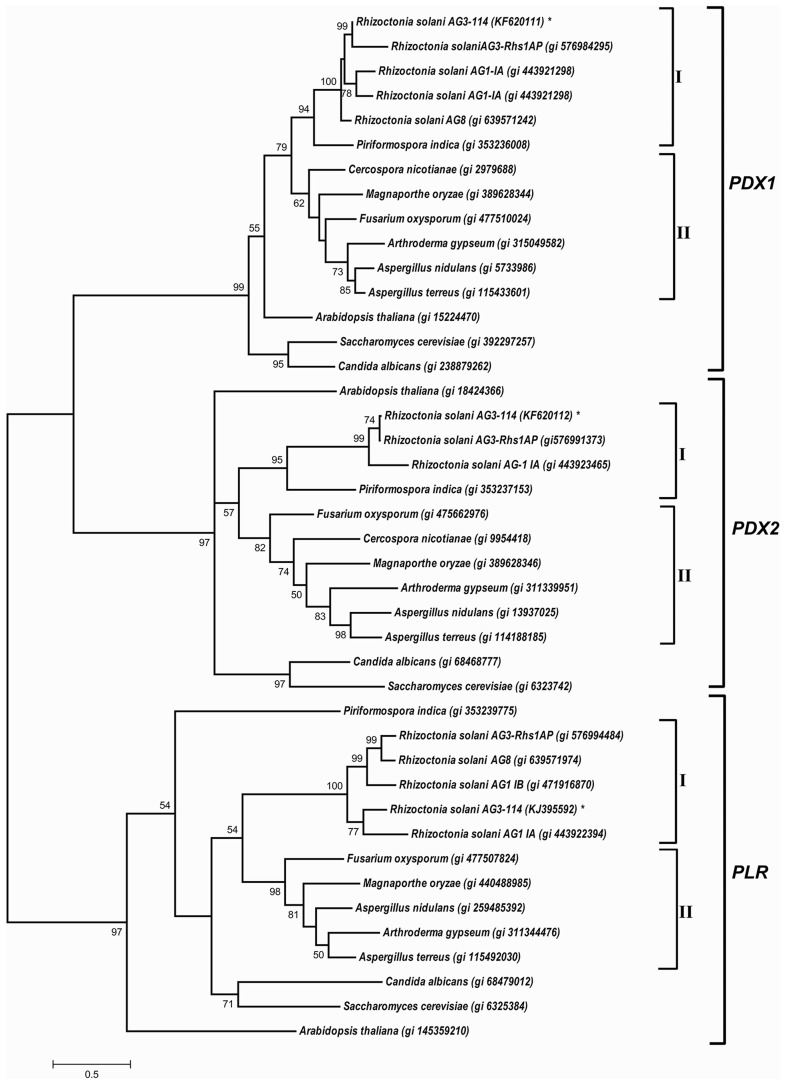
**A phylogenetic tree based on the similarity among cDNA sequences of ***PDX1***, ***PDX2***, and ***PLR*** from selected fungi and plants**. The phylogenetic relationship was generated from multiple sequence alignments using Clustal Omega and the tree was constructed using the program MEGA 6.0, and generated by the maximum likelihood method (Tamura et al., 2011). NCBI accession numbers are listed next to the species names. Asterisk indicates *R. solani* AG3- 114 cDNA sequences of *RsolPDX1, RsolPDX2, and RsolPLR*. Bootstrap scores (1000 replicates) are shown at nodes only when they are higher than 50%. I: Basidiomycetes, II: Ascomycetes.

The phylogenetic tree of *PDX1, PDX2*, and *PLR* shows a complete separation between the three families of vitamin B6 biosynthesis genes. Within each gene family, Basidiomycete, and Ascomycete sequences have clustered in separate clades. *Candida albicans* and *Saccharomyces cerevisiae* were clustered in a separate clade for each gene. *PDX1, PDX2*, and *PLR* sequences of different *R. solani* anastomosis groups are consistently clustered together in a separate clade (Figure 6). Within each of the two families, *RsolPDX1/*2 are most closely related to *PDX1*/*2* of Rhs1AP, another strain of *R. solani* AG3. Finally, within the aldo-keto reductase superfamily, *RsolPLR* gene is closely clustered to the *PLR* gene of *R. solani* AG1-IA, the rice sheath blight pathogen (Figure 6).

### *R. solani* genes of the vitamin B6 *de novo* and *Salvage* pathways are differentially regulated by different stress inducers

To provide insight into the regulation of *R. solani* vitamin B6 biosynthesis genes exposed to different stress inducers, and investigate whether they could play a role in homeostasis of ROS, the relative transcript abundances of *RsolPDX1, RsolPDX2*, and *RsolPLR* genes were assessed in *R. solani* at 24 and 72 HPT with paraquat and PAA and also at 0.5 and 3 HPT with H_2_O_2_.

#### Paraquat

**Figure 7:**
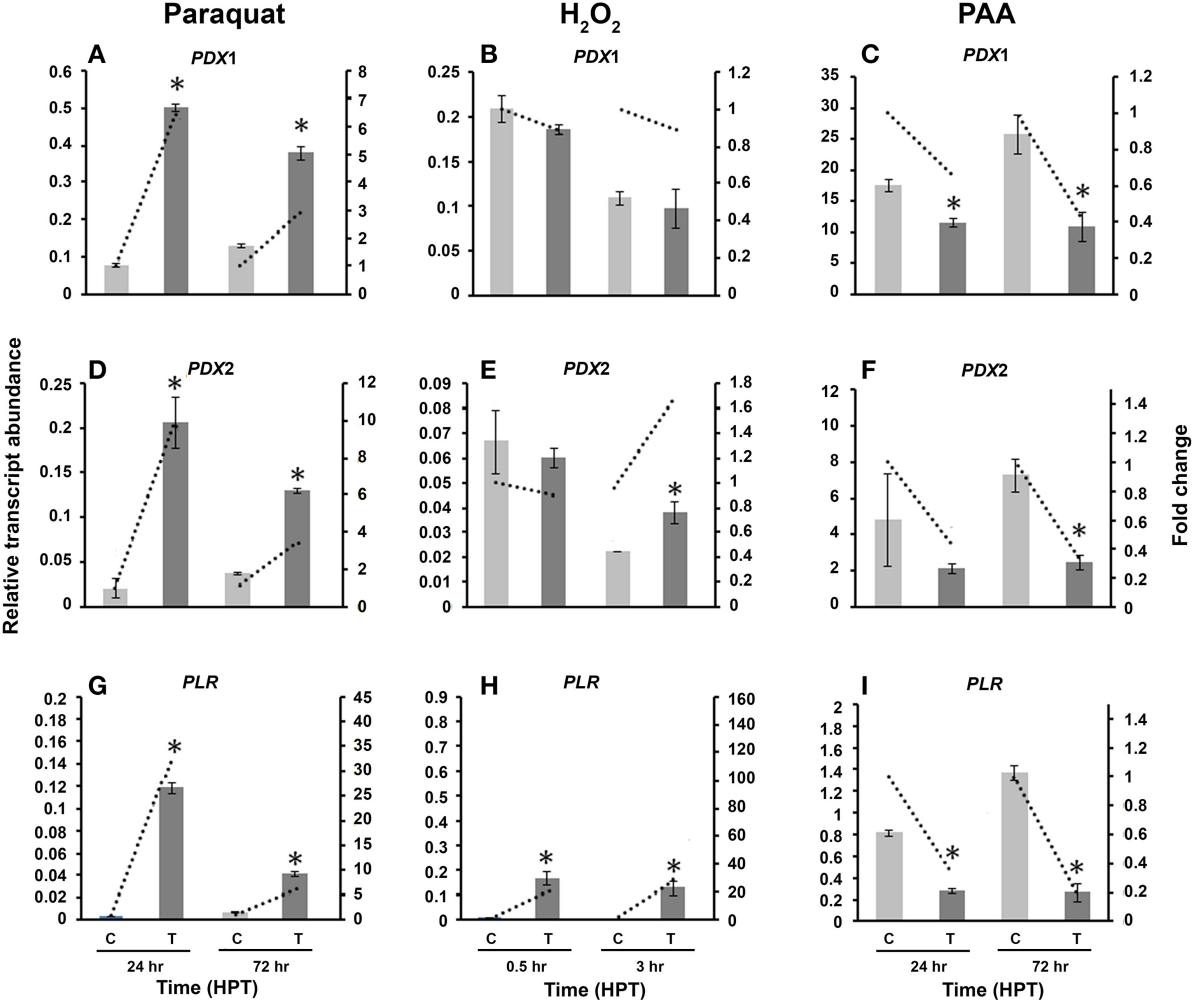
**Effect of abiotic stress on ***R. solani*** AG3 vitamin B6 genes**. Regulation of *RsolPDX1* **(A–C)**, *RsolPDX2* **(D–F)**, and *RsolPLR* **(G–I)** transcripts when grown in PDB amended with paraquat (4 mM) **(A,D,G)**, hydrogen peroxide (H_2_O_2_) (5 mM) **(B,E,H)** or phenylacetic acid (PAA) (7.5 mM) **(C,F,I)**. The relative transcript abundance of gene expression was normalized with appropriate housekeeping genes (G3PDH for paraquat and H_2_O_2_; Histone and Tubulin for PAA). C, control; *R. solani* grown without stress inducers. T, treatment with stress inducer. Asterisk indicates significant relative transcript abundance between the control and interaction at each time point using Least Significant Difference test (*P* < 0.05). Bars represent the average relative transcript abundance of three biological replicates ± standard deviation. Dotted line represents fold change which was calculated by normalization of treatment samples with appropriate controls at each corresponding time point.

Compared to control cultures, the relative transcript abundance of *RsolPDX1* and *RsolPDX2* has significantly increased at both time points with a higher fold increase of 6.4 and 9.9 in *RsolPDX1* and *RsolPDX2*, 24 HTP, respectively (Figures 7A,D). Likewise, there was a significant increase in relative transcript abundance level of *RsolPLR* at both HPT with a notable fold increase of 32.8 (Figure 7G).

#### H_2_O_2_

Exposure of *R. solani* mycelia to H_2_O_2_ had no effect on *RsolPDX1* (Figure 7B) but an effect on *RsolPDX2* at 3 HPT with a slight fold increase (1.68 fold; Figure 7E). Importantly H_2_O_2_ caused a notable increase in *RsolPLR* relative transcript abundance at 0.5 and 3 HPT with a substantial fold increase of 27.8 fold at 3 HPT (Figure 7H).

#### PAA

To obtain further insights into the putative activities of vitamin B6, we also assessed whether the relative transcripts abundance of *RsolPDX1, RsolPDX2*, and *RsolPLR* are regulated by PAA, an antimicrobial and antioxidant compound produced by *R. solani* AG3 (Bartz et al., 2012). The three genes were mostly down-regulated in the range of 5.0–1.5 fold at 24 and 72 HPT, respectively (Figures 7C,F,I).

### The antioxidant encoding genes *GST* and *Catalase* are transcriptionally regulated in *R. solani* by abiotic stress

**Figure 8:**
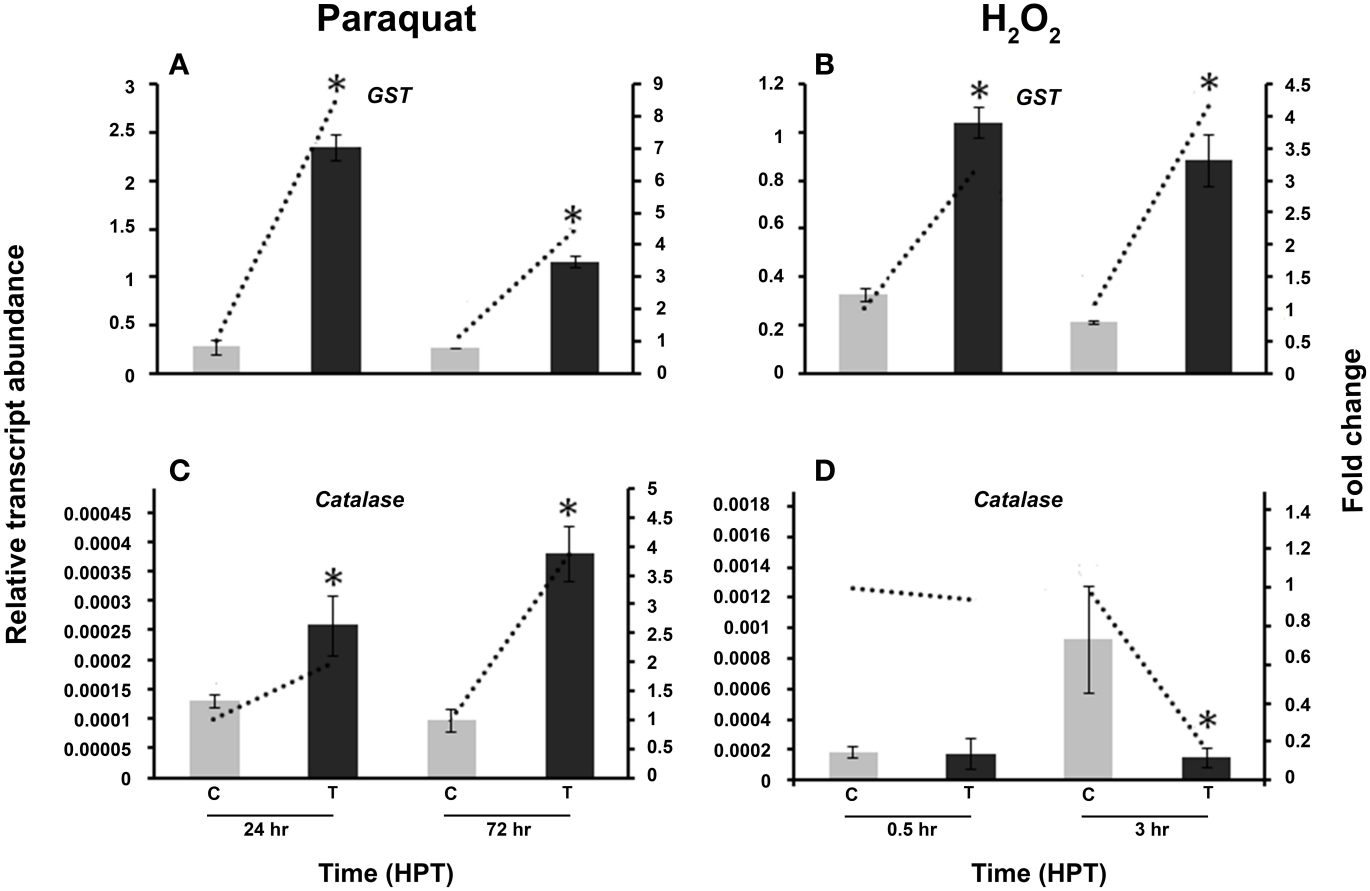
**Effect of abiotic stress on ***R. solani*** AG3 ***glutathione S-Transferase*** (***GST***) and ***catalase*** genes**. Regulation of *R. solani* AG3 *GST* **(A,B)**, and *catalase* **(C,D)** transcripts when grown in PDB amended with paraquat (4 mM) **(A,C)** or hydrogen peroxide (H_2_O_2_) (5 mM) **(B,D)**. The relative transcript abundance of gene expression was normalized with G3PDH. C, control; *R. solani* grown without stress inducers. T, treatment with stress inducer. HPT, hours post treatment. Asterisk indicates significant relative transcript abundance between the control and interaction of each time point using Least Significant Difference test (*P* < 0.05). Bars represent the average relative transcript abundance of three biological replicates ± standard deviation. Dotted line represents fold change which was calculated by normalization of treatment samples with appropriate controls at each corresponding time point.

To confirm the presence of oxidative stress in *R. solani*, the activity of other well-known antioxidant genes like *GST* and *catalase* was compared to the activity of the vitamin B6 genes under the same abiotic stress conditions (Figure 8). *GST* was significantly induced by paraquat and H_2_O_2_ (Figures 8A,B) with an 8.6 fold and 4.2 increase at 24 and 72 HPT, respectively. Paraquat significantly upregulated *catalase* at both time periods, with a highest increase of 3.9 fold at 72 HPT (Figure 8C). However, exposure to H_2_O_2_ caused a downregulation of 5.9 fold decrease as compared to the control at 3 HPT (Figure 8D).

### *R. solani* vitamin B6 genes are regulated by external supplementation of vitamin B6 vitamers

**Figure 9:**
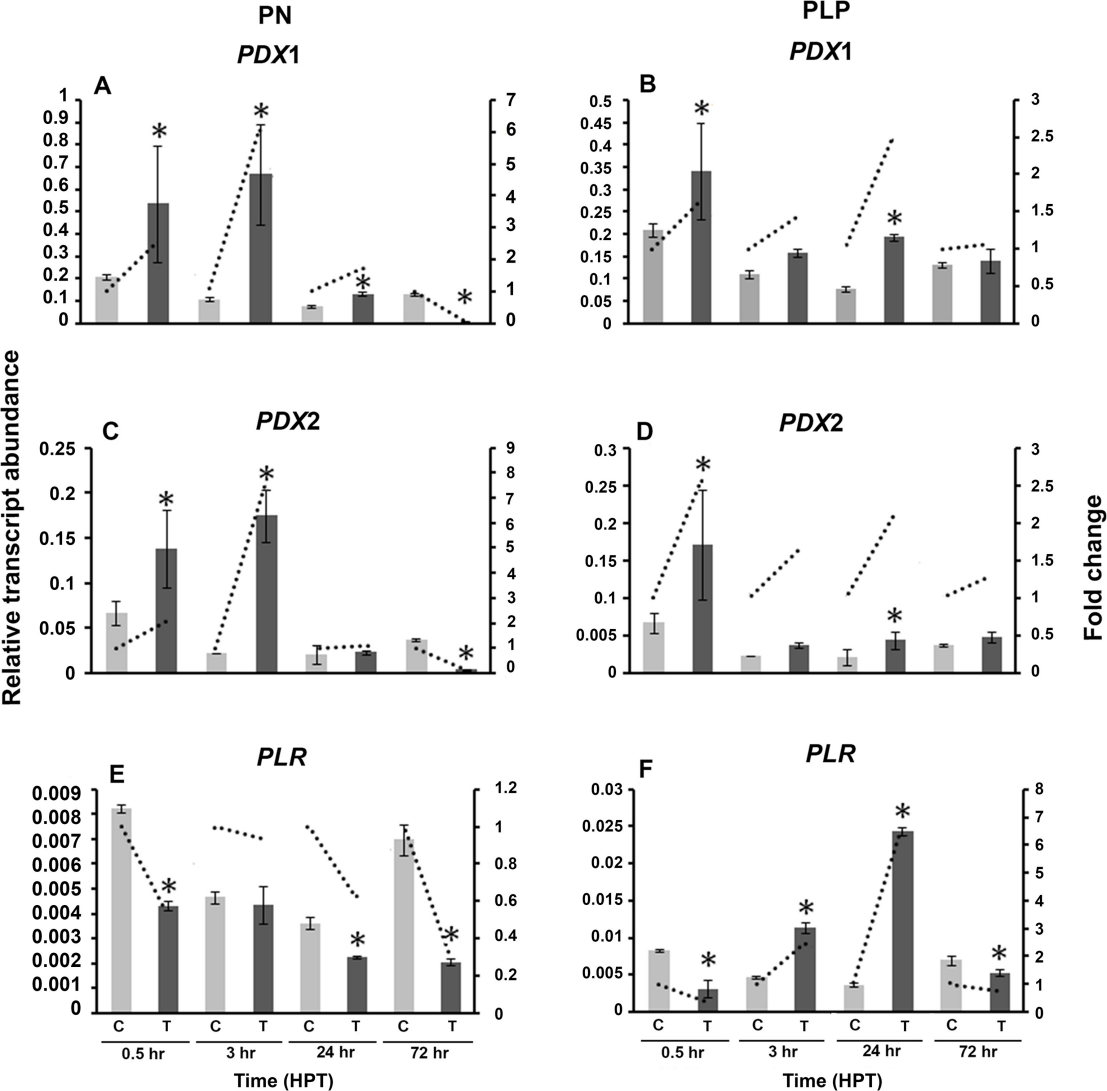
**Effect of Vitamin B6 vitamers (PN: pyridoxine or PLP: pyridoxal 5′-phosphate) on ***R. solani*** AG3 vitamin B6 genes**. Regulation of *RsolPDX1* **(A,B)**, *RsolPDX2* **(C,D)**, and *RsolPLR* **(E,F)** transcripts when grown in PDB amended with PN (0.01 g/L) **(A, C, E)** or PLP (0.01 g/L) **(B, D, F)**. The relative transcript abundance of gene expression was normalized with G3PDH. C, control; *R. solani* grown without stress inducers. T, treatment with vitamers. HPT, hours post treatment. Asterisk indicates significant relative transcript abundance between the control and interaction of each time point using Least Significant Difference test (*P* < 0.05). Bars represent the average relative transcript abundance of three biological replicates ± standard deviation. Dotted line represents fold change which was calculated by normalization of treatment samples with appropriate controls at each corresponding time point.

To understand the role of vitamin B6 vitamers on the transcriptional regulation of vitamin B6 genes, *R. solani* was grown in PDB amended with either PN or PLP. No growth differences were observed between the control and the treatments (data not shown). Exogenous addition of PN, induced the regulation of both *RsolPDX1* and *RsolPDX2* (6.1 and 7.7 fold, respectively) at 3 HPT (Figures 9A,C), caused a drop to near basal level values at 24 HPT (1.7 and 1.1 fold, respectively), followed by significant down-regulation (16.7 and 9.1 fold, respectively) at 72 HPT (Figures 9A,C). PN caused a decrease in *RsolPLR* relative transcript abundance at all time points (Figure 9E). The amendment of growth medium with PLP, induced the expression of *RsolPDX1* and *RsolPDX2* at 24 HPT followed by a drop to the basal level at 72 HPT (Figures 9B,D). There was a substantial increase in transcript relative abundance of *RsolPLR* 3 HPT (2.5 fold) and 24 HPT (6.7 fold) when PLP was added exogenously (Figure 9F).

### Accumulation of ROS in *R. solani* mycelia is linked to transcriptional regulation of *RsolPDX1, RsolPDX2*, and *RsolPLR*

**Figure 10:**
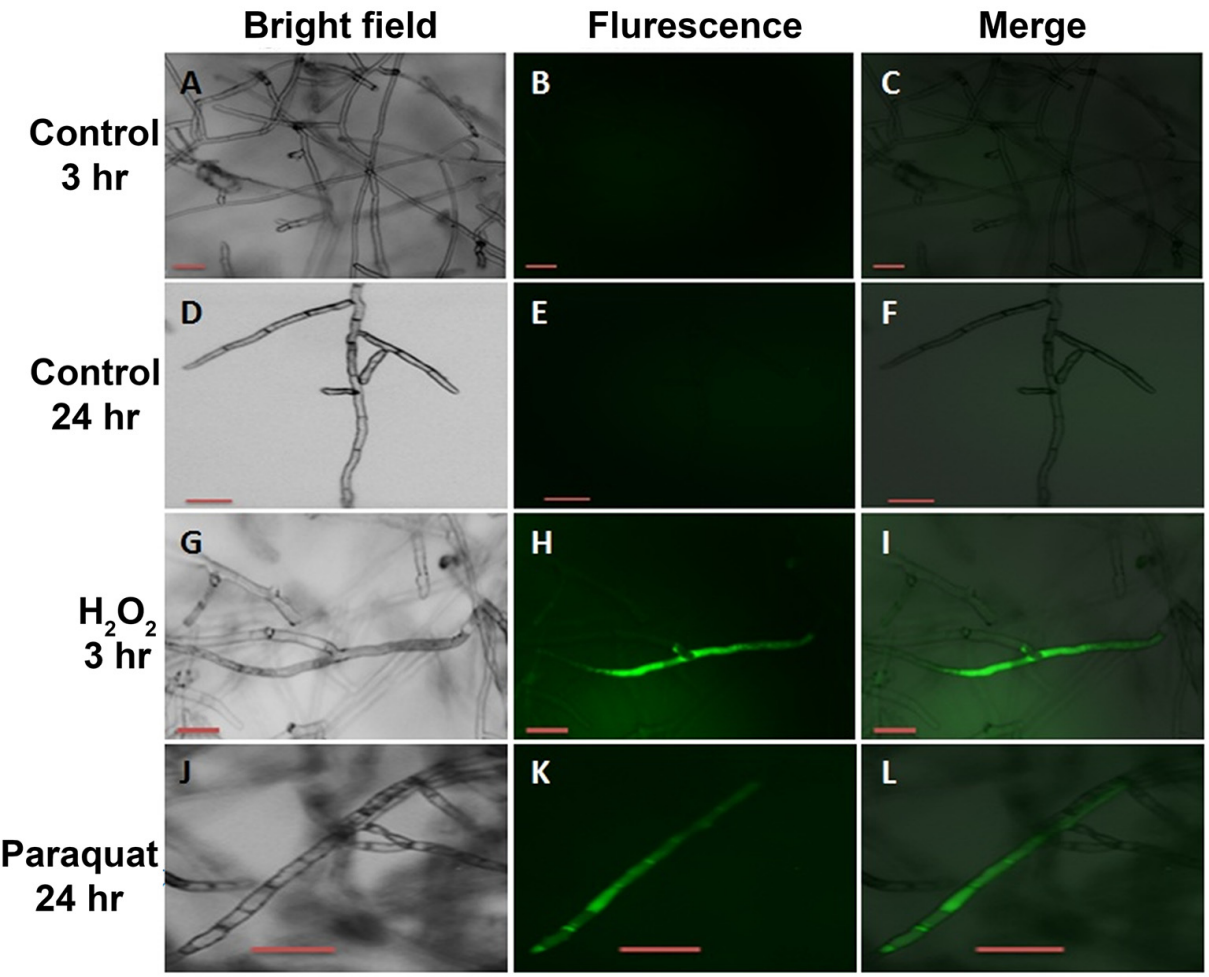
**Intracellular production of ROS in ***R. solani*** AG3 mycelia grown for 3 days before exposure to 4 mM paraquat or 5 mM H_**2**_O_**2**_**. The production of ROS was visualized by fluorescence microscopy using the ROS-sensitive probe H2DCF-DA. Control mycelia with no evidence of endogenous ROS production at 3 HPT **(A–C)** or at HPT 24 **(D–F)**. Mycelia grown in PDB amended with 5 mM H_2_O_2_ at 3 HPT **(G–I)** or 4 mM paraquat at 24 HPT **(J–L)**. Bar = 50 μm.

**Figure 11:**
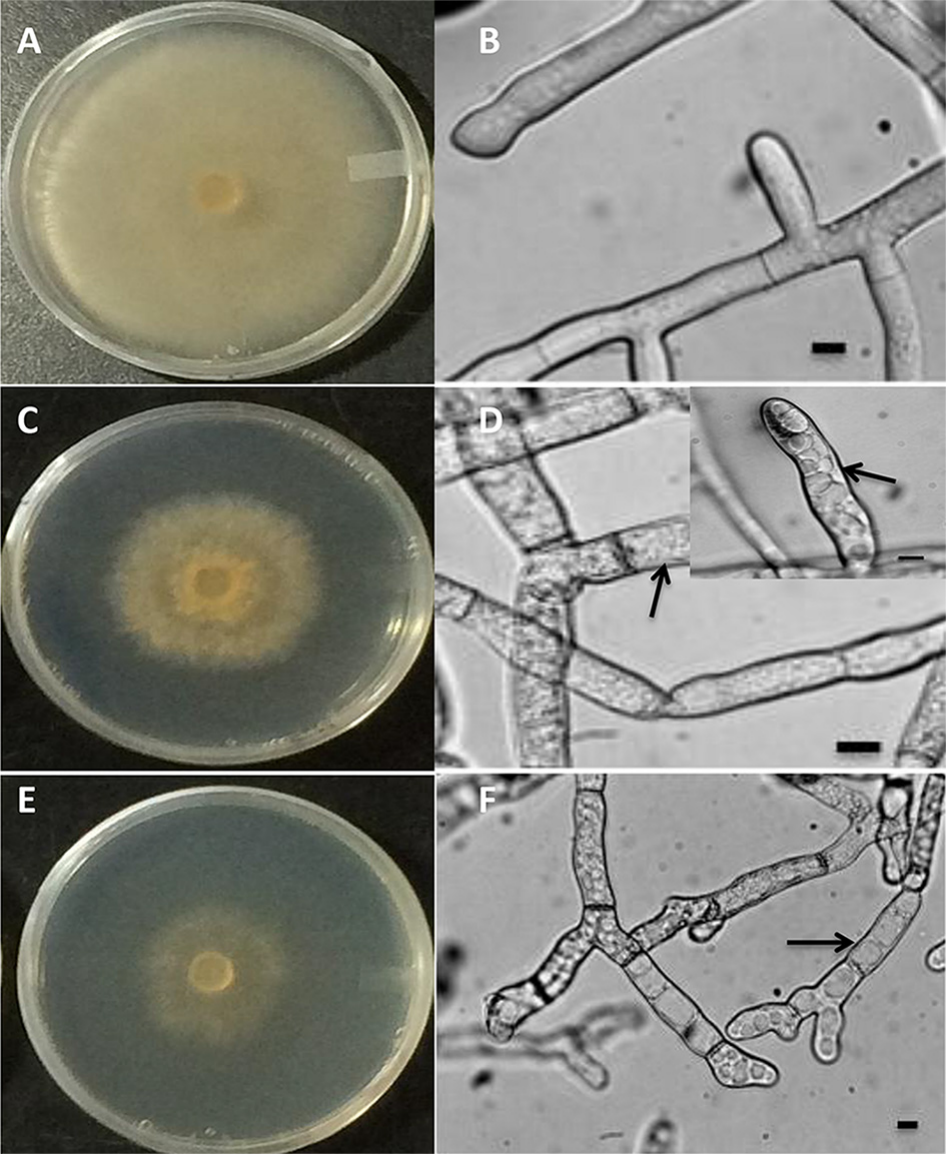
**Macroscopic (A,C,E) and microscopic (B,D,F) images of ***R. solani*** AG3 mycelia grown for 3 days before exposure to abiotic stress**. Control mycelia in PDB at 3 HPT or at 24 HPT showing normal hyphal cellular organization **(A,B)**. Mycelia grown in PDB amended with 5 mM H_2_O_2_ at 3 HPT (**C,D**, inset) or 4 mM paraquat at 72 HPT **(E,F)** displaying intracellular disorganization, vacuolarization, and loss of cytoplasm (arrows). Bar = 10 μm.

To determine whether the increase in vitamin B6 transcripts in the paraquat or H_2_O_2_-treated mycelia are linked to an accumulation of ROS in the fungal hyphae, the presence of these chemical species was estimated by adding H2DCF-DA to the growing media in which the fungus was exposed to 3 and 24 h under H_2_O_2_ and paraquat stress, respectively. An intense green fluorescence, corresponding to the oxidative form of H2DCF-DA, was detected in both treatments (Figures 10G–L). Hyphae of the untreated control plates did not display any green fluorescence (Figures 10A–F). *R. solani* growth was reduced by the addition of paraquat and H_2_O_2_ as compared to the control treatment (Figures 11A,C,E). Additionally, H_2_O_2_ and paraquat-treated hyphae showed increased levels of vacuolarization and compaction (Figures 11D,F) when compared to the control (Figure 11B) leading to loss of viability. These data confirm that exposure of the fungus to paraquat or H_2_O_2_ induces an oxidative stress in the fungal hyphae and could be related to the oxidative status of the fungus.

## Discussion

In this study, we characterized for the first time the *PDX1/PDX2* genes of the *de novo* component and the *PLR* gene of the salvage component of the vitamin B6 biosynthetic pathway in the plant pathogenic fungus *Rhizocotnia solani* AG3 (Rs114) and provided the evidence for their transcriptional regulation under different stress inducers. We also show that these genes might play a role in oxidative stress protection in *R. solani*.

Sequences of the vitamin B6 genes are similar to those found in other organisms. The predicted amino acid sequences of the three genes showed high degree of similarity to other fungal PDX1, PDX2, and PLR proteins, and their close relatedness to other fungal homologs was confirmed (Ehrenshaft et al., 1998; Nakano et al., 1999; Ehrenshaft and Daub, 2001; Ellis, 2002; Barski et al., 2008; Benabdellah et al., 2009). The release of the draft genome sequence of *R. solani* AG3 (strain Rhs1AP) (Cubeta et al., 2014) further confirmed the genes' annotations. The presence of conserved amino acids suggests the ability of PDX1-PDX2 complex formation that is crucial for vitamin B6 biosynthesis (Belitsky, 2004; Raschle et al., 2005). Interestingly, there is variation in the number of introns present in various vitamin B6 *de novo* biosynthesis genes. As opposed to the *PDX1* or *PDX2* homologs identified in *C. nicotianae* and *Glomus intraradices* which are intronless (Ehrenshaft et al., 1998; Ehrenshaft and Daub, 2001; Benabdellah et al., 2009), the three vitamin B6 genes in *R. solani* AG3 (Rs114) contain introns similar to *PyroA* (*PDX1*) gene of *Aspergillus nidulans* (Osmani et al., 1999). From an evolutionary perspective, the presence of introns is advantageous because it provides organisms with diversity of proteins through alternative splicing or because introns might possess elements that are involved in regulation of gene expression (Kalsotra and Cooper, 2011; Yang et al., 2013).

Southern blot analysis indicated that two copies are present for *RsolPDX1* and *RsolPLR*, most likely as a result of gene duplication, and a single copy is present for *RsolPDX2* in *R. solani* AG-3 (Rs114). While most other fungi contain a single copy of those genes (Ehrenshaft et al., 1998; Osmani et al., 1999; Ehrenshaft and Daub, 2001; Benabdellah et al., 2009), *S. cerevisiae, SNZ* (*PDX1* homolog), and *SNO* (*PDX2* homolog) genes were shown to have three members each with only *SNZ1* and *SNO1* being implicated in vitamin B6 biosynthesis (Padilla et al., 1998; Rodríguez-Navarro et al., 2002). Only one copy of each of the three genes was found in the draft genome sequence of *R. solani* AG3 (strain Rhs1AP). This could be an indication that the *PDX1, PDX2*, and *PLR* gene copy numbers differ among the various *R. solani* strains, although this will require subsequent validation once higher quality genome assemblies are released. Our Southern hybridization provides only preliminary evidence of gene copy number. We don't exclude the possibility of having additional copies due to high molecular size of the fragments obtained. Much remains to be determined whether the two copies of *RsolPDX1* and *RsolPLR* are both functional or one of them is a pseudogene.

To date, evidence that *R. solani* genes of the vitamin B6 pathway are upregulated in response to biotic stress has been reported (Morissette et al., 2008; Chamoun and Jabaji, 2011; Gkarmiri et al., 2015). However, to the best of our knowledge, little is known of the physiological responses of *R*. *solani* to various oxidative stress and also no studies exist on transcriptional regulation of genes in the vitamin B6 biosynthetic pathway, nor on their role in oxidative stress alleviation in *R. solani* experiencing abiotic stress.

The increased formation of ROS in hyphal cells can induce oxidative stress and damage to DNA, RNA, protein, and lipids, leading to the loss of cell viability (Apel and Hirt, 2004; Sharma et al., 2012). Our results clearly showed that paraquat and H_2_O_2_ induced ROS formation in hyphal cells of *R. solani* leading to growth reduction and loss of viability of hyphal cells. These observations were linked to the regulation of vitamin B6 encoding genes and their collective possible role as oxidative stress alleviation. Our findings indicate exposure of *R. solani* to paraquat upregulates *RsolPDX1* and *RsolPDX2* expression possibly suggesting a role of vitamin B6 in protection of *R. solani* against superoxide anions. In other organisms, gene regulation studies have continually connected vitamin B6 to oxidative stress and noted an increased transcriptional activity of vitamin B6 *de novo* pathway genes in response to oxidative stress. As examples, *GintPDX1*, the *Glomus intrarradices* and SNZ1, the *S. cerevisiae* PDX1 homolog and PDX2 of *S. pombe* showed increased transcript accumulation upon treatments with paraquat and H_2_O_2_, respectively (Lee et al., 1995; Chen et al., 2003; Benabdellah et al., 2009).

Furthermore, we show evidence that the vitamin B6 salvage pathway appears to be involved in the oxidative stress response in *R. solani*. *RsolPLR* transcript levels were up-regulated by both paraquat and H_2_O_2_ at all time points. This finding is in agreement with the transcriptional increase of *PLR* in *S. pombe* after 1 h of H_2_O_2_ treatment (Chen et al., 2003) and with the role of *PLR* in PN catalysis (Morita et al., 2004; Sang et al., 2007). The reason for the differential transcriptional regulation of *RsolPDX1* and *RsolPDX2* and *RsolPLR* by H_2_O_2_ and superoxide radicals is unknown, and may reflect differences in the type of damage induced by ROS.

To overcome oxidative damage, all living organisms have developed antioxidant systems to efficiently quench ROS excess and to keep ROS production and scavenging systems in check (Sharma et al., 2012). Maintaining cellular ROS homeostatis is achieved by the production of non-enzymatic antioxidants such as *GST* and enzymatic antioxidants such as catalases. Therefore, studying the regulation of *GST* and *catalase* in stressed cultures of *R. solani* is direct confirmation that these genes play a role in oxidative stress protection. Similar to vitamin B6 encoding genes, differential upregulation of *GST* and *catalase* was observed in response to the type of stressor, although the fold increase in vitamin B6 encoding genes was substantially higher. These results may indicate that vitamin B6, via the expression of *RsolPDX1* and *RsolPDX2* and *RsolPLR*, has better ability than glutathione to decrease ROS levels (Ehrenshaft et al., 1999).

The role of *RsolPDX1* and *RsolPDX2* and *RsolPLR* in vitamin B6 biosynthesis suggests a possible regulation of their expression by vitamin B6 and the expression of the homologs is dependent on vitamin B6 availability. Surprisingly in other biological systems, regulation of *PDX1* expression was not affected by the addition of vitamin B6 (Rodríguez-Navarro et al., 2002; Benabdellah et al., 2009). The authors claimed that this independence is related to the high constitutive expression levels that may mask the effect of the absence of the vitamin. In our study, both PN and PLP vitamers up-regulated the vitamin B6 *de novo* genes, *RsolPDX1*, and *RsolPDX2*, in *R. solani*. Given the involvement of the pyridoxine encoding *de novo* genes in vitamin B6 biosynthesis, and the central role of PLP as a cofactor, a regulatory switch based on the amount of PN and PLP is likely to exist (Mooney et al., 2009), it is very plausible that vitamin B6 vitamers may act as regulators. Alternatively, the addition of vitamers might be perceived by *R. solani* as signal indicating stress leading to upregulation of the *de novo* vitamin B6 genes at the onset of treatment. In a similar fashion, plants supplemented with the well-known antioxidants riboflavin (vitamin B2) or thiamine (vitamin B1) developed systemic resistance to bacterial or fungal infections along with increased transcription of PR related genes (Dong and Beer, 2000; Ahn et al., 2005). As for the decrease in the transcript abundance levels of both *RsolPDX1* and *RsolPDX2* at 72 HPT might be due to conversion of PN into PNP and eventually to PLP, resulting in decrease of substrate availability (Zhao and Winkler, 1995; Rueschhoff et al., 2012). In this regard mechanisms of how vitamers regulate the *de novo* vitamin B6 genes remain to be explored further.

Contrary to *RsolPDX1* and *RsolPDX2*, the transcriptional response of *RsolPLR* was vitamer specific; a down regulation in response to PN and up regulation in response to PLP. These results were expected since PN is the product of the reaction catalyzed by *PLR* (Herrero et al., 2011) causing a decrease in relative transcript abundance of *RsolPLR*. Increase in transcript levels may be due to the conversion of PLP to PL, the substrate for PLR (Zhao and Winkler, 1995; Herrero et al., 2011).

## Conclusion

*RsolPDX1, RsolPDX2*, and *RsolPLR* represent three important components of the vitamin B6 pathway in *R. solani* AG3 (Rs114). These genes are differentially regulated upon various types of oxidative stress, such as paraquat and H_2_O_2_. Taken together, the indirect participation of the *de novo* (*RsolPDX1* and *RsolPDX2*) and salvage (*RsolPLR*) vitamin B6 genes in the oxidative stress response of the plant pathogenic fungus *R. solani* is strongly suggested. In yeasts, gene disruption and over expression have been valuable in delineating the biological role of *PDX1* in fungi. Comparable approaches should prove equally valuable to define the role of *R. solani* vitamin B6 genes.

## Author contributions

JS, RC, and SJ conceived, designed, and executed the experiments. EG helped in the execution of certain experiments. JS and RC analyzed the data. JS, RC, and SJ contributed to the writing of the manuscript.

### Conflict of interest statement

The authors declare that the research was conducted in the absence of any commercial or financial relationships that could be construed as a potential conflict of interest.
